# Barriers to physical activity among university students in the light of psychosocial and body composition determinants

**DOI:** 10.1186/s40359-025-03789-4

**Published:** 2025-12-03

**Authors:** Mustafa Akil

**Affiliations:** Faculty of Sport Sciences, Uşak University, Uşak, Türkiye

**Keywords:** Physical activity barriers, Fatigue, Motivation, Body composition

## Abstract

**Background:**

Physical activity is essential for maintaining a healthy lifestyle; however, various psychological and physiological barriers can hinder participation. University students represent a high-risk group due to academic workload and social pressures. This study aimed to investigate barriers to physical activity participation among university students in the context of fatigue, motivation, and body composition determinants.

**Methods:**

The study employed a cross-sectional and observational design, and a total of 552 individuals with a mean age of 20.5 ± 2.1 years were included in the analysis. Data were collected through a demographic information form, the Barriers to Physical Activity Scale, the Chalder Fatigue Scale, and the University Student Motivation Scale. Body composition was assessed using a bioelectrical impedance analysis device. In addition to descriptive statistics, Pearson’s correlation analysis was used to examine relationships between variables, and multiple linear regression was applied to determine the effects of predictor variables.

**Results:**

Higher levels of fatigue were associated with increased perceptions of both personal (*r* ≈ .28) and social barriers (*r* ≈ .21), indicating that fatigue is a significant psychological constraint on physical activity participation. While intrinsic motivation demonstrated a protective effect (β=–0.22), extrinsic motivation (β = 0.19) and amotivation (β = 0.27) were linked to heightened perceptions of barriers. Furthermore, higher body fat percentage emerged as the strongest physiological predictor (β = 0.29), underscoring the critical role of body composition in shaping behavioural barriers.

**Conclusion:**

In conclusion, barriers to physical activity among university students are influenced by both psychosocial dynamics and body composition characteristics. Strategies aimed at enhancing intrinsic motivation, while reducing fatigue and body fat, may substantially contribute to overcoming barriers in this age group.

## Introduction

Regular physical activity plays a critical role in reducing obesity and preventing chronic diseases [1]. Moreover, exercise exerts restorative effects on the musculoskeletal system, producing favourable changes in biomarkers associated with muscle damage [2]. Numerous studies have demonstrated that physical activity lowers the risk of prevalent chronic conditions such as cardiovascular disease, diabetes, and cancer [3]. In addition, participation in regular activity among obese individuals has been shown to substantially reduce the risk of all-cause mortality [4]. According to the World Health Organization, more than 80% of adolescents fail to achieve the recommended level of physical activity [5]. This situation highlights the persisting risk particularly for young adults. University students, in particular, represent one of the most vulnerable groups to insufficient activity due to increasing academic pressures and sedentary lifestyles [6]. Therefore, promoting physical activity in young populations constitutes a strategic priority not only for individual health but also for public health.

Research conducted in various countries has shown that a considerable proportion of university students are physically inactive. For instance, a study in Jordan reported that fewer than half of the students engaged in regular physical activity, and this was associated with variables such as obesity and perceived health status [7]. Similarly, large-scale studies across Europe have found that approximately half of students do not meet the recommended activity levels [8]. In the literature, the most frequently cited reasons for this low activity include lack of time, insufficient facilities, academic workload, and reduced motivation. A systematic review by Brown et al. (2024) revealed that time constraints, social influences, and lack of prioritisation were the most influential factors shaping students’ activity behaviours [9]. Likewise, Silva et al. (2022) emphasised in their review that time, motivation, and accessibility were the most prevalent barriers among both high school and university students [10]. In Türkiye, empirical evidence similarly indicates that physical inactivity among university students is widespread; studies consistently show that nearly two-thirds of students fail to meet recommended activity levels, and that perceived barriers are strongly associated with leisure-time management, academic pressure, and limited access to adequate facilities [11, 12]. Moreover, recent research from Türkiye has highlighted that students’ insufficient activity is closely linked to psychosocial determinants such as low motivation, irregular daily routines, and inability to allocate time for exercise, suggesting a culturally specific pattern of barrier perception. These findings indicate that insufficient physical activity may have negative consequences not only for health but also for academic achievement and psychological well-being [13]. Therefore, initiatives aimed at increasing physical activity among university students can be considered critical for both individual health and academic performance.

Among university students, fatigue resulting from academic and social demands represents a major barrier to participation in physical activity. In particular, lack of time and low energy levels often lead individuals to relegate physical activity to a secondary priority [10]. This contributes to the spread of physical inactivity during young adulthood and may establish behavioural patterns that negatively affect health in the long term [9]. Motivational processes play a critical role in overcoming such barriers. According to Self-Determination Theory, when individuals’ basic psychological needs are fulfilled, their intrinsic motivation is strengthened, enabling them to maintain behaviours in a more sustainable manner [14]. SDT conceptualises motivation as a continuum ranging from autonomous forms (intrinsic motivation) to controlled forms (external and introjected regulation), and finally to amotivation. This hierarchical structure suggests that as individuals move from autonomous toward controlled motivation, their sense of volition decreases and behaviour becomes increasingly dependent on external pressure or reward. Within this framework, autonomous forms of motivation have been shown to enhance physical activity participation, whereas controlled or extrinsic motivation tends to be limited to short-term engagement [15]. Because extrinsic motivation is driven by external demands, social expectations, or avoidance of guilt rather than genuine personal interest, it is theorised to increase perceived barriers by reducing enjoyment, internal commitment, and long-term adherence. Students who act under controlled motivation often experience conflict between imposed demands and personal preferences, which may further elevate perceptions of time pressure, fatigue, and environmental constraints. Recent studies conducted with university students support this theoretical perspective. Findings suggest that intrinsic motivation and satisfaction derived from sport are positively associated with higher levels of physical activity, while extrinsic motivation and amotivation are linked to increased perceptions of barriers [16]. Similarly, research among physiotherapy students has identified fatigue and physical strain as significant barriers, with low motivation reported to be associated with reduced activity levels [17]. Taken together, these findings indicate that both fatigue and motivational factors need to be addressed simultaneously in order to sustain physical activity habits among students.

Among university students, high body fat percentage and low muscle mass represent some of the most significant physiological barriers to physical activity participation [18]. Students with greater muscle mass and a higher fat-free mass index demonstrate advantages in terms of daily step count and overall activity level, whereas those with higher fat percentages tend to participate less in physical activity [19]. Evidence also indicates that obesity and overweight in students are associated with lower activity levels and limited cardiorespiratory fitness [20]. Excess fat mass not only reduces physical capacity but also leads to impairments in metabolic functions [21]. Therefore, obesity in students should not be viewed merely as an issue of aesthetics or excess weight but as a barrier directly related to performance and health. On the other hand, exercise interventions have been found effective in improving body composition. Systematic reviews have demonstrated that aerobic and high-intensity interval training can reduce body fat percentage while increasing muscle mass [22]. Collectively, this evidence suggests that considering body composition variables alongside psychosocial factors may provide a more comprehensive understanding of physical activity habits among university students [7].

University students constitute one of the most vulnerable groups in terms of regular physical activity participation due to both academic and social pressures [9]. This situation has negative consequences not only for health but also for academic achievement and psychological well-being [6, 10]. Yet, young adulthood is a critical life stage in which lifelong health behaviours are formed, and the habits acquired during this period directly influence later risks such as obesity, cardiovascular disease, and metabolic syndrome [4]. Existing studies in the literature have largely focused on single-dimensional barriers (e.g., only environmental factors or only motivation), while integrative approaches that examine the interaction of different determinants (such as fatigue, motivation, and body composition) remain limited. This gap underscores the need to develop more comprehensive models to explain students’ physical activity behaviours. In this context, the present study focuses specifically on psychosocial (fatigue and motivation) and physiological (body composition) determinants of perceived barriers to physical activity, and all analyses were conducted using only these variables. No additional psychological constructs (e.g., depression, anxiety, stress) were measured or included in any regression model. Accordingly, the aim of this study is to investigate barriers to physical activity participation among university students by simultaneously examining fatigue, motivation, and body composition indicators. The research hypotheses were formulated in line with the variables actually included in the analyses: H_1_: fatigue levels will increase perceived barriers; H_2_: intrinsic motivation will act as a protective factor, whereas extrinsic motivation and amotivation will increase risk; H_3_: higher body fat percentage will increase barriers while greater muscle mass will reduce them; and H4: in multivariate regression models using fatigue, motivation dimensions, and body composition variables as predictors, intrinsic motivation, fatigue, and body fat will emerge as independent predictors. By addressing psychosocial and physiological factors together, this study seeks to provide a more comprehensive understanding of physical activity barriers among university students and to generate empirical evidence for strategies that support both health and academic performance.

## Methods

### Study design and ethical approval

This research was designed as a cross-sectional and observational study. Ethical standards applicable to scientific research involving human participants were observed, and all procedures were conducted in accordance with the most recent principles of the Declaration of Helsinki. Prior to participation, all volunteers were provided with a detailed explanation of the study’s purpose, scope, and potential risks, after which written informed consent was obtained. The study protocol was approved by the Ethics Committee of Uşak University (Decision No:2025 − 281).

### Sample size calculation

Prior to the study, the adequacy of the sample size was tested using G*Power 3.1.9.4 software. Calculations were based on an F test (Linear multiple regression: R² deviation from zero), with parameters set to represent a small effect size (f² = 0.02), a significance level of α = 0.05, and a statistical power of 1 − β = 0.80. An a priori analysis indicated that a minimum of 550 participants would be sufficient to detect small effects in the planned regression models. The final sample consisted of 552 students, thus meeting and slightly exceeding the required sample size derived from the a priori analysis. In accordance with the reviewer’s recommendation, post hoc power values were not used for inferential interpretation, as post hoc power based on observed R² offers limited methodological value.

### Inclusion criteria and demographic characteristics

The study group consisted of undergraduate students enrolled at universities in Türkiye. The inclusion criteria were: being 18 years of age or older, having active student status, and signing an informed consent form. Exclusion criteria included a history of acute illness, diagnosed psychiatric disorder, pregnancy, and substance dependence. These criteria were established because such health conditions could directly influence the study variables (namely, physical activity level, fatigue, motivation, and body composition) and thereby introduce potential bias into the results. Due to time and resource limitations, a convenience sampling method was used, and no probabilistic or random selection procedures were applied. A total of **552** students participated in the study, of whom 51.1% were female (*n* = 282) and 48.9% were male (*n* = 270). In terms of age distribution, 58.2% were aged 20 years or younger (*n* = 321), 35.5% were between 21 and 23 years (*n* = 196), and 6.3% were 24 years or older (*n* = 35). Examination of the mean values revealed that participants’ body weight was 67.6 ± 14.3 kg, height 171.2 ± 9.6 cm, skeletal muscle mass 29.30 ± 7.38 kg, and body mass index 23.01 ± 3.89 kg/m². All body composition measurements were successfully obtained from all participants.

### Study design and procedure

The study was designed as a cross-sectional investigation conducted among university students. Using a convenience sampling method, 600 students were approached through classroom announcements, campus postings, and online student communication channels; of these, 552 participants who met the inclusion criteria and completed all assessments without missing data were included in the analysis. Prior to analysis, the dataset was examined for missing or inconsistent responses, and records not meeting the criteria were excluded using the listwise deletion method. Data collection was carried out face-to-face under standardised conditions. At the outset, a pilot study was conducted with a small group of students to test the feasibility of the data collection instruments. Feedback obtained during this process was used to confirm the clarity of the questionnaires and the appropriateness of the administration order. All assessments were performed in the same physical setting, under fixed environmental conditions, and between 09:00–12:00, thereby minimising circadian rhythm–related physiological variability [23, 24]. Participants were instructed to fast for at least three hours prior to measurement, to empty their bladder, and to refrain from vigorous exercise, alcohol, or excessive caffeine consumption during the preceding 24 h. Data collection followed a three-stage protocol. In the first stage, participants’ demographic and lifestyle information (e.g., age, sex, housing conditions, dietary habits) was recorded. In the second stage, psychosocial assessments were conducted: fatigue was measured using the Fatigue Scale, barriers to physical activity with the Barriers to Physical Activity Participation Scale, and motivation with the University Student Motivation Scale. In the third stage, body composition was assessed, including body weight, height, body mass index, skeletal muscle mass, and body fat percentage. All questionnaires and measurements were administered in a single session, following a fixed order and conducted by trained researchers. The devices used were regularly calibrated according to the manufacturers’ instructions. To ensure data reliability, all responses were anonymised, recorded using identification codes, and entered through a double data entry process. This approach aimed to enhance measurement reliability and maintain the internal validity of the study.

### Data collection instruments and scales

#### Barriers to physical activity participation scale

The Barriers to Physical Activity Participation Scale, used to evaluate perceived barriers to physical activity among university students, was originally developed by Ibrahim et al. (2013) as a 24-item instrument comprising three subscales [25]. The scale was later adapted into Turkish and validated by Yurtçiçek et al. (2018) [26]. In the Turkish adaptation, the scale was structured as a 22-item instrument scored on a five-point Likert format (1 = strongly disagree, 5 = strongly agree). Higher scores indicate a greater perceived level of barriers. Factor analysis supported the three-factor structure, with a Kaiser–Meyer–Olkin coefficient of 0.84 and a statistically significant Bartlett’s test of sphericity [χ²(231) = 2346.96], demonstrating sample adequacy. The scale consists of three subscales: Personal (14 items), Social Environment (3 items), and Physical Environment (5 items). The possible score range for the total scale is 22–110, with subscale score ranges of 14–70 (Personal), 3–15 (Social Environment), and 5–25 (Physical Environment). In the original study, internal consistency was reported as Cronbach’s α = 0.85 for the overall scale and α = 0.68–0.74 for the subscales. For the Turkish version, Cronbach’s α was reported as 0.87 for the total scale, 0.85 for the Personal subscale, 0.53 for the Social Environment subscale, and 0.72 for the Physical Environment subscale. The administration time of the scale is approximately 5–7 min. These findings demonstrate that the Turkish version of the scale is a reliable and valid tool for assessing factors that limit participation in physical activity [25, 26].

#### Chalder fatigue scale

In this study, the revised 11-item form of the Chalder Fatigue Scale (CFS-11) was used to assess participants’ fatigue levels. The original version of the scale was developed by Chalder et al. (1993) and was revised by Cella and Chalder in 2010 through the removal of three items [27, 28]. The CFS-11 comprises two subscales: Physical Fatigue (CFS-PF) and Mental Fatigue (CFS-MF). In the present study, the Likert-type scoring system was used, consisting of a four-point response format (0 = never, 1 = more than usual, 2 = much more than usual, 3 = very much more than usual). Using this scoring method, the possible score ranges are 0–21 for Physical Fatigue, 0–12 for Mental Fatigue, and 0–33 for the total fatigue score, with higher scores indicating greater levels of perceived fatigue. The cultural adaptation and psychometric evaluation of the scale in Türkiye was conducted by Adın et al. (2022) [29]. Their findings confirmed that the two-factor structure of the Turkish version was preserved, internal consistency was high (CFS total α = 0.863; CFS-PF α = 0.862; CFS-MF α = 0.704), and test–retest reliability was at a good level (ICC = 0.76). Construct validity analyses demonstrated excellent model fit (CFI = 0.963, RMSEA = 0.06, SRMR = 0.02), and criterion validity supported the use of a ≥ 12-point cut-off to distinguish individuals with elevated fatigue levels. Validity analyses further demonstrated that the CFS correlated with other relevant measures as expected, discriminated effectively between known groups, and showed high specificity in distinguishing fatigued individuals using a ≥ 12-point cut-off. The scale requires approximately 2–4 min to administer, and these results indicate that the Turkish version of the CFS-11 is a reliable and valid instrument for measuring perceived fatigue in both clinical and community-based research [27–29].

#### University student motivation scale (USMS)

In order to assess the motivation levels of university students, the University Student Motivation Scale (USMS) was employed. Developed by Yılmaz (2018) on the basis of Self-Determination Theory, the scale was designed to capture motivational dynamics in university populations in Türkiye [30]. The USMS consists of 26 items rated on a 4-point Likert-type scale (1 = strongly disagree, 4 = strongly agree), with higher scores indicating stronger endorsement of the corresponding motivational construct. Its three-dimensional structure—intrinsic motivation, extrinsic motivation, and amotivation—was confirmed through factor analyses; the KMO value was reported as 0.89, and the scale explained 53.5% of the total variance. The possible score ranges for each subscale are as follows: intrinsic motivation (16 items; 16–64), extrinsic motivation (5 items; 5–20), and amotivation (5 items; 5–20). Reliability analyses indicated strong internal consistency, with Cronbach’s alpha coefficients ranging from 0.65 to 0.93 and approximately 0.87 for the total scale. Test–retest results further demonstrated temporal stability, with correlation coefficients ranging from *r* =.56–0.77 across subscales and approximately *r* =.88 for the total score. Criterion-related validity analyses showed strong and theoretically expected associations with the Academic Motivation Scale (AMS), including positive correlations between the intrinsic motivation subscales of both instruments (*r* ≈.79), moderate associations for extrinsic motivation (*r* ≈.57), and meaningful relationships for amotivation (*r* ≈.62). The interrelations between USMS subscales were also theoretically coherent, with positive intrinsic–extrinsic motivation associations, negative intrinsic–amotivation associations, and non-significant extrinsic–amotivation associations. The administration time of the USMS is approximately 3–5 min, and its inclusion of social and communicative motivation items provides broader coverage of motivational processes in higher education settings. Collectively, these findings demonstrate that the USMS is a valid and reliable instrument for assessing university students’ motivation levels [30].

### Bioelectrical impedance analysis

Participants’ body composition was assessed using a bioelectrical impedance analysis (BIA) device, the InBody 120 (InBody Co., Seoul, Korea) [31]. This device applies the direct segmental multi-frequency BIA (DSM-BIA) method and performs a total of 10 impedance measurements at 20 kHz and 100 kHz across five body segments: right arm, left arm, trunk, right leg, and left leg. No empirical prediction equations are used; instead, calculations are based directly on the impedance values. The validity and reliability of InBody devices have been demonstrated in numerous studies through comparative analyses with gold-standard methods such as Dual-Energy X-ray Absorptiometry (DXA). DSM-BIA systems report high correlations (*r* =.85–0.95) in estimating fat-free mass and fat mass and exhibit high internal consistency and test–retest reliability [32]. All measurements were conducted under standardised pre-assessment conditions: participants were instructed to remain fasting for at least three hours, to empty their bladder, and to avoid vigorous exercise, alcohol, or excessive caffeine for the preceding 24 h. Before measurement, participants stood barefoot on the device platform, removed all metal accessories, and remained in an upright posture with arms and legs slightly abducted to ensure accurate current flow. A resting period of approximately 3–5 min was provided prior to assessment to stabilise hydration-related fluctuations. To ensure measurement reliability, repeated assessments were conducted on 10% of the participants, with no significant differences observed between the first and second measurements. All measurements were performed by trained researchers using identical procedures, and device calibration was routinely checked according to manufacturer guidelines.

### Statistical methods

The data obtained from the study were analysed using SPSS version 26.0. The distributional characteristics of the variables were examined using the Kolmogorov–Smirnov test, and skewness and kurtosis values within the acceptable range (–2 to + 2) were taken as additional indicators of approximate normality, in line with recommendations for large samples. Means and standard deviations were calculated to summarise the data. The reliability of the scales was assessed using internal consistency coefficients; Cronbach’s α values were 0.854 for the Fatigue Scale, 0.907 for the Barriers to Physical Activity Scale, and 0.877 for the Motivation Scale, indicating satisfactory reliability in the present sample. Pearson’s correlation analysis was used to examine bivariate associations between variables, and correlation coefficients were interpreted using Cohen’s effect size conventions. Multiple linear regression analyses were conducted to identify the predictors of physical activity barriers. Four regression models were constructed: (1) fatigue dimensions, (2) motivation subscales, (3) body composition variables, and (4) a combined multivariate model including fatigue, motivation, and body composition indicators. All dependent and independent variables used in each model corresponded directly to the constructs measured in this study; no additional psychological variables (e.g., depression, anxiety, stress) were included. Before conducting the regression analyses, assumption checks were performed, including linearity, normality of residuals, homoscedasticity, and the detection of multivariate outliers using Cook’s distance. Multicollinearity was examined using Variance Inflation Factor (VIF) values, and all reported models satisfied the criterion of VIF < 5. Model fit was evaluated using the F statistic, and explanatory power was presented using R² and adjusted R² values. For all statistical procedures, a significance level of *p* <.05 was adopted.

## Results

According to the data presented in Table 1, the students’ mean fatigue score was 2.27 ± 0.58. Among the subscales, physical fatigue was higher (2.35 ± 0.72), while mental fatigue was lower (2.18 ± 0.59). This indicates that participants experienced more physical fatigue than mental fatigue. The mean total score for barriers to physical activity was 2.19 ± 0.71; among the subscales, the highest barrier was related to the physical environment (2.26 ± 0.88), whereas the lowest was related to the social environment (2.16 ± 0.90). This suggests that students perceived inadequacies in physical facilities and environments as greater barriers. The mean motivation score was 1.24 ± 0.42, with intrinsic motivation being the highest (1.65 ± 0.62) and amotivation the lowest (0.75 ± 0.68). These findings reveal that students were generally dominated by intrinsic motivation, although some exhibited tendencies towards amotivation. In terms of body composition, the participants’ mean skeletal muscle mass was 29.30 ± 7.38 kg, mean body fat percentage was 22.38 ± 9.30, and mean basal metabolic rate was 1500.74 ± 256.44 kcal. These values suggest that students were generally within normal ranges, although a wide variation was observed in body fat percentages (Table 1).

**Table 1:** Mean scores of the questionnaires and body composition analysis

	*N*	Mean	SD
Fatigue (Total)	552	2.27	0.58
Physical Fatigue	552	2.35	0.72
Mental Fatigue	552	2.18	0.59
Barriers to Physical Activity (Total)	552	2.19	0.71
Personal	552	2.17	0.74
Social Environment	552	2.16	0.90
Physical Environment	552	2.26	0.88
Motivation (Total)	552	1.24	0.42
Intrinsic Motivation	552	1.65	0.62
Extrinsic Motivation	552	1.33	0.62
Amotivation	552	0.75	0.68
Skeletal Muscle Mass (kg)	552	29.30	7.38
Body Fat Percentage %	552	22.38	9.30

As shown in Table 2, the total score for barriers to physical activity demonstrated significant but generally small-to-moderate associations with fatigue, motivation, and body composition variables. Total fatigue, physical fatigue, and mental fatigue were all positively correlated with barrier scores, with correlations ranging from small to moderate effect sizes (*r* =.239–0.351). Intrinsic motivation showed small negative correlations with all barrier types (*r* = −.119 to − 0.156), indicating that students with higher intrinsic motivation tended to perceive slightly fewer barriers. By contrast, extrinsic motivation demonstrated negligible correlations, reaching significance only for social environment barriers (*r* =.087), and should therefore be interpreted cautiously. Amotivation exhibited small but significant positive correlations with all barrier categories (*r* =.156–0.192), suggesting that increases in amotivation were consistently associated with higher perceived barriers. Regarding body composition, skeletal muscle mass and basal metabolic rate both showed small negative correlations with barrier scores (*r* ≈ −.17 to − 0.29), whereas body fat percentage demonstrated small to moderate positive correlations (*r* =.174–0.326). This pattern suggests that while fatigue and body fat were the most notable correlates of perceived barriers, these associations remained modest in magnitude. Overall, the correlation analysis indicates that although several psychosocial and physiological factors are statistically related to barriers to physical activity, the effect sizes are predominantly small, underscoring the need for cautious interpretation and suggesting that multiple determinants collectively contribute to students’ perceptions of physical activity barriers (Table 2).

**Table 2:** Pearson correlations between barriers to physical activity and psychosocial and body composition variables

	BPA-Total	Personal	Social	Physical
Fatigue (Total)	0.351** (medium)	0.351** (medium)	0.302** (medium)	0.239** (small)
Physical Fatigue	0.305** (medium)	0.327** (medium)	0.270** (small)	0.182** (small)
Mental Fatigue	0.314** (medium)	0.288** (small)	0.261** (small)	0.246** (small)
Motivation (Total)	0.068 (negligible)	0.073 (negligible)	0.029 (negligible)	0.073 (negligible)
Intrinsic Motivation	− 0.144** (small)	− 0.119** (small)	− 0.156** (small)	− 0.087* (negligible)
Extrinsic Motivation	0.070 (negligible)	0.087* (negligible)	0.032 (negligible)	0.062 (negligible)
Amotivation	0.192** (small)	0.162** (small)	0.164** (small)	0.156** (small)
SMM (kg)	− 0.268** (small)	− 0.287** (small)	− 0.176** (small)	− 0.222** (small)
BF (%)	0.268** (small)	0.326** (medium)	0.174** (small)	0.193** (small)

*BPA* Barriers to Physical Activity, *SMM* Skeletal Muscle Mass, *BF* Body Fat Percentage, *BMR* Basal Metabolic Rate

* *p* <.05, ** *p* <.01

Correlations with |r| < 0.10 were considered negligible; 0.10–0.29 small; 0.30–0.49 medium; ≥ 0.50 large (Cohen, 1988)

As shown in Table 3, motivation dimensions demonstrated small but statistically significant associations with all types of physical activity barriers. Intrinsic motivation emerged as a consistent negative predictor of barrier perceptions, with standardized coefficients indicating small effect sizes across personal (β = −0.204), social (β = −0.210), and physical environmental barriers (β = −0.129). These findings suggest that students who report higher intrinsic motivation tend to experience slightly fewer barriers. In contrast, extrinsic motivation showed small positive associations with all three barrier categories (β = 0.190 for personal, β = 0.138 for social, and β = 0.121 for physical environmental barriers). Although these effects were statistically significant, their magnitude remained modest, indicating that controlled forms of motivation contribute only minimally to increases in perceived barriers. Similarly, amotivation displayed small positive effects on personal (β = 0.094), social (β = 0.100), and physical environmental barriers (β = 0.113), reflecting a pattern in which higher amotivation corresponds to higher barrier perceptions. Despite these significant relationships, the explanatory power of the regression models was low (adjusted R^2^ = 3–5%), indicating that motivation variables accounted for only a small proportion of the variance in perceived barriers. Taken together, the results suggest that while motivational dynamics are statistically relevant, they represent only one of several factors influencing students’ perceptions of physical activity barriers (Table 3).

**Table 3:** Multiple regression analysis predicting barriers from motivation dimensions

Barrier Type	Predictor	B	SE	β	t	*P*	VIF	95% CI
Personal	Intrinsic Motivation	−0.243	0.064	−0.204	−3.817	< 0.001	< 2	[−0.368, −0.118]
Personal	Extrinsic Motivation	0.229	0.063	0.19	3.628	< 0.001	< 2	[0.106, 0.352]
Personal	Amotivation	0.102	0.048	0.094	2.104	0.036	< 2	[0.008, 0.196]
Social Environment	Intrinsic Motivation	−0.307	0.078	−0.21	−3.932	< 0.001	< 2	[−0.460, −0.154]
Social Environment	Extrinsic Motivation	0.203	0.077	0.138	2.634	0.009	< 2	[0.052, 0.354]
Social Environment	Amotivation	0.133	0.059	0.1	2.248	0.025	< 2	[0.017, 0.249]
Physical Environment	Intrinsic Motivation	−0.183	0.077	−0.129	−2.389	0.017	< 2	[−0.334, −0.032]
Physical Environment	Extrinsic Motivation	0.173	0.076	0.121	2.285	0.023	< 2	[0.024, 0.322]
Physical Environment	Amotivation	0.145	0.058	0.113	2.509	0.012	< 2	[0.031, 0.259]

Effect size interpretation follows Cohen’s (1988) benchmarks for coefficient of determination (R²): small ≈ 0.02, medium ≈ 0.13, and large ≈ 0.26. The regression models in this study fall within the small-to-medium range, indicating modest explanatory power despite statistically significant predictors

As shown in Table 4, both physical and mental fatigue significantly predicted students’ perceived barriers to physical activity. For personal barriers, physical fatigue (β = 0.242, *p* <.001) and mental fatigue (β = 0.155, *p* =.001) were both significant positive predictors, indicating that higher fatigue levels were associated with increased perceptions of personal limitations. A similar pattern emerged for social environment barriers, with both physical fatigue (β = 0.181, *p* <.001) and mental fatigue (β = 0.162, *p* =.001) contributing significantly to the model. In contrast, physical environment barriers were predicted only by mental fatigue (β = 0.208, *p* <.001), while the effect of physical fatigue was non-significant (β = 0.068, *p* =.168). The explanatory power of the models was modest but meaningful, with fatigue dimensions accounting for 12% of the variance in personal barriers, 9% in social environment barriers, and 6% in physical environment barriers. Overall, these results indicate that mental fatigue plays a more consistent role across all types of barriers, whereas physical fatigue primarily influences personal and social limitations but not environmental constraints (Table 4).

**Table 4:** Multiple regression analysis predicting barriers from fatigue dimensions

Barrier Type	Predictor	B	SE	β	t	*P*	VIF	95% CI
Personal Barriers	Physical Fatigue	0.249	0.049	0.242	5.076	< 0.001	< 2	[0.153, 0.345]
Personal Barriers	Mental Fatigue	0.193	0.06	0.155	3.25	0.001	< 2	[0.075, 0.311]
Social Environment	Physical Fatigue	0.227	0.061	0.181	3.719	< 0.001	< 2	[0.107, 0.347]
Social Environment	Mental Fatigue	0.247	0.074	0.162	3.339	0.001	< 2	[0.102, 0.392]
Physical Environment	Physical Fatigue	0.083	0.06	0.068	1.381	0.168	< 2	[−0.035, 0.201]
Physical Environment	Mental Fatigue	0.309	0.073	0.208	4.224	< 0.001	< 2	[0.166, 0.452]

Effect size interpretation follows Cohen’s (1988) benchmarks for coefficient of determination (R²): small ≈ 0.02, medium ≈ 0.13, and large ≈ 0.26. The regression models in this study fall within the small-to-medium range, indicating modest explanatory power despite statistically significant predictors

As shown in Table 5, body composition variables demonstrated distinct associations with perceived barriers to physical activity. Body fat percentage emerged as a significant positive predictor across all three models. For personal barriers, higher body fat percentage was strongly associated with greater perceived barriers (β = 0.634, *p* <.001), whereas skeletal muscle mass was not a significant predictor (*p* =.065). A similar pattern was observed for social environment barriers, where body fat percentage remained a significant predictor (β = 0.344, *p* =.001), while skeletal muscle mass did not contribute meaningfully to the model (*p* =.473). For physical environment barriers, body fat percentage again demonstrated a significant positive effect (β = 0.343, *p* =.001), whereas skeletal muscle mass showed no significant association (*p* =.269). The explanatory power of the models varied, accounting for 15% of the variance in personal barriers (R² = 0.151), 4% in social environment barriers (R² = 0.037), and 6% in physical environment barriers (R² = 0.065). Overall, these findings indicate that body fat percentage consistently predicts higher perceived barriers to physical activity, whereas skeletal muscle mass does not independently influence barrier perceptions (Table 5).

**Table 5:** Multiple regression analysis predicting barriers from body composition variables

Barrier Type	Predictor	B	SE	β	t	*P*	VIF	95% CI
Personal Barriers	SMM (kg)	−0.011	0.006	−0.089	−1.846	0.065	< 2	[−0.023, 0.001]
Personal Barriers	Body Fat (%)	0.036	0.005	0.634	7.124	< 0.001	< 2	[0.026, 0.046]
Social Environment	SMM (kg)	−0.005	0.007	−0.035	−0.718	0.473	< 2	[−0.019, 0.009]
Social Environment	Body Fat (%)	0.018	0.006	0.344	3.215	0.001	< 2	[0.006, 0.030]
Physical Environment	SMM (kg)	−0.007	0.007	−0.054	−1.106	0.269	< 2	[−0.021, 0.007]
Physical Environment	Body Fat (%)	0.019	0.006	0.343	3.426	0.001	< 2	[0.007, 0.031]

Effect size interpretation follows Cohen’s (1988) benchmarks for coefficient of determination (R²): small ≈ 0.02, medium ≈ 0.13, and large ≈ 0.26. The regression models in this study fall within the small-to-medium range, indicating modest explanatory power despite statistically significant predictors

**Fig. 1:**
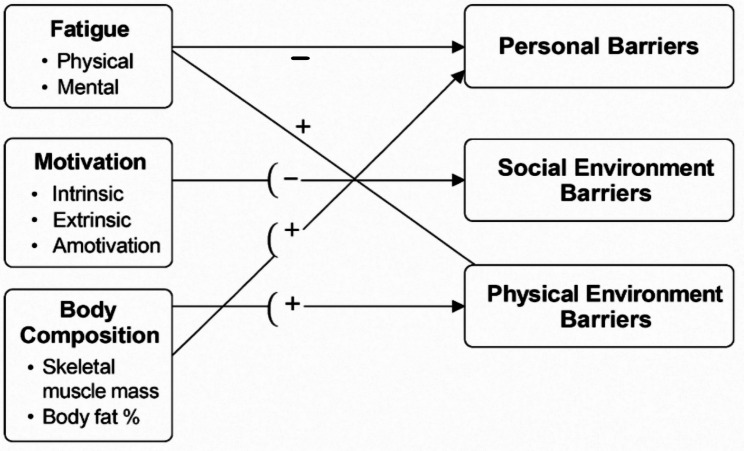
Significant psychosocial and physiological predictors of physical activity barriers

Figure 1 provides a schematic summary of our findings, illustrating how fatigue (both physical and mental), motivation (intrinsic, extrinsic, and amotivation), and body composition (skeletal muscle mass, body fat percentage) interact to influence students’ perceived barriers to physical activity. The diagram highlights the complex and multidimensional relationships identified in this study, showing protective and risk-enhancing effects across psychosocial and physiological domains.

## Discussion

Our findings indicate that both total fatigue and its subdimensions significantly increased barriers to physical activity, with mental fatigue showing a distinct and stronger contribution to the perception of environmental barriers. This pattern suggests that psychosocial burdens among university students reinforce not only personal barriers but also social and physical environmental constraints. Consistent with our results, the systematic review by Silva et al. (2022) emphasised lack of time, low motivation, and insufficient energy as the most common barriers among students-factors that closely resemble the fatigue patterns observed in the present study [10]. Brown et al. (2024) likewise showed that academic stress and energy depletion limited students’ capacity to maintain regular physical activity [9]. In Jordanian students, Alkhawaldeh et al. (2024) similarly identified fatigue and limited facilities as key correlates of low activity levels [7]. Beyond perceived energy loss, experimental findings further illustrate the behavioural effects of fatigue: Van As et al. (2021) reported that individuals evaluated physical effort as more costly following mental fatigue [33], and Shava et al. (2024) noted that students frequently viewed exercise as fatiguing and effortful [34]. Prieto-González and Alkouatli (2025) also showed that fatigue interacts with contextual barriers (such as time management and environmental conditions) to heighten perceived obstacles [35]. Taken together, the present study adds to this literature by demonstrating, in an integrated model, that mental fatigue specifically heightens perceptions of environmental barriers, a finding that has rarely been emphasised in previous research. These results underline the importance of designing interventions that address not only physical fatigue but also mental exhaustion and cognitive load, which appear to play a critical role in shaping physical activity behaviour among university students. Although body fat percentage emerged as a strong predictor (β = 0.634), this coefficient should be interpreted in the context of the model’s modest overall explanatory power, indicating that the effect represents a robust statistical association rather than a large practical impact. In line with this, the low-to-moderate R² values observed across models (6–15%) highlight that, despite statistically significant coefficients, the practical significance of these predictors is limited. These results suggest that interventions should focus on multifactorial approaches rather than assuming strong causal or high-impact effects from any single variable.

Analysis of the data revealed that intrinsic motivation was negatively associated with all types of physical activity barriers, whereas extrinsic motivation and amotivation were positively associated with stronger perceptions of these barriers. This pattern is consistent with the propositions of Self-Determination Theory, which posits that more self-determined forms of motivation support voluntary and sustained engagement in health behaviours [14]. Prieto-González et al. (2025) likewise reported that self-determined motivation enhanced long-term adherence to physical activity across different cultural contexts [35]. Similarly, Ahsan et al. (2024) observed that although motivational levels varied across demographic factors, intrinsic motivation played a more influential role in supporting regular activity [36]. Among female university students, Alajlan et al. (2024) showed that autonomy and psychological need satisfaction helped maintain motivation, whereas controlled forms of regulation undermined stability [37]. Zhong (2024) further demonstrated that intrinsic motivation was positively associated with psychological well-being, while extrinsic motivation and amotivation attenuated this link [38]. Liu et al. (2023) also found that exercise motivation supported physical fitness levels, partly through increased physical activity [39]. Taken together with our findings, these results suggest that motivational quality (rather than motivational quantity) is central to how students perceive barriers. Intrinsic motivation appears to function as a protective factor, while extrinsic motivation and amotivation align with heightened barrier perceptions, reflecting their more externally driven and less sustainable nature.

It was observed that higher body fat percentage consistently emerged as the only significant predictor of physical activity barriers among university students, whereas skeletal muscle mass did not independently predict any barrier type. This suggests that excess fat mass may increase perceptions of psychosocial and environmental barriers through its influence on physical capacity, movement efficiency, and self-efficacy. Imai and Kubo (2023) reported that students with greater lean and muscle mass demonstrated advantages in daily step count and activity levels [18]. Similarly, Musijowska and Kwilosz (2024) emphasised that higher body fat limited physical activity participation, whereas muscle mass supported regular engagement [19]. Petřeková et al. (2024) also found that obesity and overweight were associated with low cardiorespiratory fitness and insufficient activity levels [20]. In addition, Torres et al. (2023) showed that higher body fat not only reduced physical capacity but also increased symptoms of depression and anxiety, thereby reinforcing perceptions of psychosocial barriers [40]. From an intervention perspective, Zhang et al. (2023) demonstrated in an exercise-based programme with university students that regular sports activities reduced body fat percentage and improved muscle mass [41]. Likewise, Tan et al. (2023) reported that combining aerobic and resistance exercises produced favourable changes in body composition and promoted muscle hypertrophy [42]. Taken together, these results align with our findings, indicating that body fat percentage plays a central role in shaping students’ perceived barriers, while muscle mass alone does not exert a significant independent effect once other factors are controlled. Regular exercise programmes that reduce fat levels may therefore provide protective effects both physiologically and psychosocially.

Our findings demonstrate that fatigue, motivation, and body composition factors each contribute independently to students’ perceptions of barriers to physical activity. Although statistically significant, the predictive models explained only a small portion of the variance, and thus practical significance should be interpreted cautiously. Rather than interacting, these variables show distinct but complementary associations, whereby higher fatigue and higher body fat percentage are linked to greater perceived barriers, while intrinsic motivation shows a protective pattern. Consistent with this, Silva et al. (2022) identified lack of time, low motivation, and insufficient energy as the most frequently reported barriers among students [10]. These results parallel those of Brown et al. (2024), who showed that stress and energy depletion caused by heavy academic responsibilities made it difficult to sustain physical activity [9]. From a motivational perspective, Prieto-González et al. (2025) reported that self-determined forms of motivation enhanced long-term adherence to activity, supporting the inverse association observed between intrinsic motivation and barrier perceptions [35]. Ahsan et al. (2024) likewise demonstrated that intrinsic motivation was a key determinant of regular activity among university students [36]. In line with these findings, Top and Akil (2021) showed that motivation to participate in sport plays a central role in shaping positive behavioural outcomes, underscoring how motivational quality can influence individuals’ engagement patterns and potentially their perceptions of constraints [43]. Furthermore, Top and Akil (2019) highlighted that emotional and social characteristics within the family context strongly affect whether young individuals are directed toward sport, suggesting that broader psychosocial environments may also shape motivation and thus perceived barriers [44]. Body composition findings add a physiological dimension: while Imai and Kubo (2023) found that students with greater muscle mass were more advantaged in terms of daily step counts [18], Torres et al. (2023) reported that higher body fat reduced physical capacity and increased psychosocial strain, potentially contributing to higher perceived barriers [40]. Taken together, these patterns suggest that psychosocial factors (fatigue and motivation) and physiological factors (body fat percentage) each play a modest but meaningful role in shaping physical activity barriers. When interpreted within the context of Türkiye, the present findings align with a growing body of national research showing that university students consistently report time constraints, academic workload, limited facilities, and motivational barriers as primary obstacles to physical activity. Studies by Kasırga et al. (2021) and Kundakcı et al. (2024) have similarly categorised student barriers under personal (time management, fatigue), social (lack of peer support), and environmental factors (facility accessibility), mirroring the three dimensions evaluated in the current study. This consistency suggests that barrier structures among Turkish students may reflect culturally embedded patterns related to academic intensity, campus infrastructure, and social norms surrounding exercise [10, 12]. The unique contribution of our study lies in examining these determinants simultaneously, allowing for a more holistic understanding of how multiple domains relate to students’ perceptions of barriers.

One of the main strengths of this study is its large sample size (*n* = 552), which increases statistical power and provides more stable parameter estimates within a heterogeneous student population. Another strength lies in the integrated analytical approach, which simultaneously examined psychosocial factors (fatigue and motivation) and physiological indicators (body composition). Previous studies in Türkiye and internationally often evaluated these domains separately, whereas the present design offers a more comprehensive perspective. Methodological clarity was further enhanced by the use of an a priori power analysis and standardised procedures for body composition assessment, supporting the reliability of physiological measurements. Despite these strengths, several limitations should be acknowledged. First, the cross-sectional design does not permit causal inference; therefore, the identified associations should be interpreted as correlational rather than directional. Second, although the sample size was large, the use of convenience sampling may limit generalisability due to potential selection bias. Third, reliance on self-report questionnaires introduces the possibility of recall and social desirability biases. Although body composition was objectively measured, physical activity behaviour itself was not assessed using objective devices, meaning that perceived barriers may not fully reflect actual activity patterns. Another limitation concerns covariate adjustment. Although variables such as age, sex, academic workload, and socioeconomic status could theoretically influence perceptions of physical activity barriers, they were not included as covariates because they were not measured with validated tools in the present dataset, and their correlations with the main study variables were weak. As such, the regression models were intentionally kept parsimonious, focusing on the primary psychosocial and physiological predictors. Finally, although body fat percentage emerged as a strong statistical predictor, the practical significance of this association should be interpreted cautiously given that the overall explanatory power of the models remained modest. Future research should address these limitations by incorporating objective measurements of physical activity (e.g., accelerometers or wearable activity monitors) to complement self-report assessments. Socioeconomic indicators, academic workload, and living arrangements should also be measured using validated instruments to better capture contextual influences on physical activity barriers. Longitudinal or experimental studies are needed to determine whether the psychosocial and physiological predictors identified in the present study exert sustained or causal effects over time, particularly in university settings in Türkiye where academic pressure and environmental constraints may play a unique role.

## Conclusion

This study demonstrates that barriers to physical activity among university students arise from a combination of psychosocial and physiological factors. Fatigue, particularly mental fatigue, was positively associated with personal, social, and environmental barriers, whereas intrinsic motivation showed a protective association. In contrast, extrinsic motivation and amotivation were linked to stronger perceived barriers. Among body composition variables, only body fat percentage emerged as a significant predictor, although the overall explanatory power of the models remained modest. Although statistically significant, the predictive models explained only a small portion of the variance, and thus their practical significance should be interpreted cautiously. These findings suggest that perceptions of barriers are shaped by both students’ motivational resources and their physical condition. Initiatives that strengthen intrinsic motivation and support students’ energy regulation may help mitigate these barriers, while interventions promoting healthy nutrition and regular exercise could contribute to more favourable body composition. Future longitudinal and experimental studies are needed to determine whether such strategies produce sustained reductions in perceived barriers.

## Data Availability

The datasets used and/or analysed during the current study are available from the corresponding author on reasonable request.
